# Teacher Becoming Curriculum Designer: Professional Teaching and Learning in China’s Early Childhood Education

**DOI:** 10.3389/fpsyg.2022.873044

**Published:** 2022-06-29

**Authors:** Xiaoming Tian, Li Bao, Tianxin Li, Yue Gu

**Affiliations:** ^1^School of Humanities and Foreign Languages, Xi’an University of Posts and Telecommunications, Xi’an, China; ^2^School of Sociology and Population Studies, Nanjing University of Posts and Telecommunications, Nanjing, China; ^3^School of Foreign Studies, Xi’an University, Xi’an, China; ^4^Department of Psychology, University of Chinese Academy of Sciences, Beijing, China

**Keywords:** Curriculum Design Coherence Model, teaching practices, China, China’s Early Childhood Education, knowledge

## Abstract

The Curriculum Design Coherence Model (CDC Model) was created as a universal curriculum design method to connect disciplinary knowledge to teachers’ expertise in a bid to promote professional teaching and learning. However, research into how the CDC Model has been adopted and localized in the Chinese educational context is scarce. This article focuses on the application and impact of the CDC Model on the resulting teaching practices in China’s Early Childhood Education (ECE) settings. The data collected through a focus group discussion with 21 teachers from a model kindergarten at the municipal level in China reveals that the CDC Model has increased the teachers’ professionalism by promoting their curriculum initiative, forging curriculum knowledge orientation, strengthening the conceptual structure within the kindergarten-developed curriculum, and enhancing the coordination between the curricula of the different courses offered by the kindergarten. This positive influence has also helped the teachers bridge their disagreement on curriculum content and pedagogy and overcome some difficulties in using the CDC Model. The study has implications for revitalizing the value of disciplinary knowledge and for viewing ECE teachers as active professional agents in ECE curriculum design and teaching.

## Introduction

The Curriculum Design Coherence Model (CDC Model) was first developed in 2017 to address the New Zealand curriculum context in the first instance and then started to be used in the “knowledge-enriched curriculum project” carried out in both New Zealand and the United Kingdom as a universal curriculum design method (Rata, 2019). The project was mainly committed to fulfilling four tasks. First, at the level of global curriculum ideology, in response to the prevailing 21st-century educational narratives which overemphasize generic skills, such as interpersonal communication and problem-solving strategies and thus ignore the importance of knowledge (Bolstad, 2012; Scott, 2015), this project proposed a knowledge-led approach to schooling (Rata, 2019, 2021b). Second, at the level of pedagogic practices, the project took disciplinary knowledge as the core of schooling, rather than students’ subjective interests, practical needs, and future workplace expectations (McPhail, 2016). Third, at the level of evaluation, the project treated the competencies developed based on the acquisition of disciplinary knowledge as the focus of curriculum evaluation and abandoned the modular assessment dominated by students’ performance (McPhail, 2017). Fourth, in terms of curriculum effects, the project aimed to explore curriculum design that can effectively link the acquisition of disciplinary knowledge with students’ cognitive architecture, thereby promoting in-depth learning and real intellectual development (Sweller et al., 2019; McPhail, 2020a).

At the very beginning of 2021, the CDC Model was introduced to a Chinese kindergarten to guide its kindergarten-based curriculum planning and teaching. Therefore, this study examines the application of the CDC Model in China’s Early Childhood Education (ECE) practices and its effectiveness. The country’s educational system includes five tiers, namely, ECE/Kindergartens (including six semesters contained in three academic years, ECE Year 1 to ECE Year 3), Primary School (Year 1-Year 6), Junior High School (Year 7-Year 9), Senior High School (Year 10-Year 12), and higher education. Among them, Primary School and Junior High School are compulsory. Although the ECE is not compulsory, its running is under the guidance of the unified nationally prescribed ECE policy (MoE, 2012).

We investigated the implementation of the CDC Model in China for two major reasons. First, an exploration of specialized databases such as Web of Science and Scopus finds that there is a dearth of research into the application of the CDC Model, as a novel and universal curriculum design method in China’s educational context, especially in the ECE setting. Second, China is a centralized country with top-down educational policies and practices (Huang et al., 2016), thereby providing a platform to navigate how the CDC Model was localized throughout the expression, adoption, and development of the country’s ECE curricula and the following teaching practices and their effects. We introduce the CDC Model first because the structure of the model provides us with both the theoretical and empirical tools to examine its application in the Chinese ECE context.

## The Curriculum Design Coherence Model

The CDC Model aims to promote professional teaching and learning by connecting disciplinary knowledge to teachers’ expertise. By separating disciplinary knowledge from sociocultural knowledge, the CDC Model argues that the acquisition of disciplinary knowledge should be the purpose of schooling (Rata, 2019, 2021b). Following the Durkheimian tradition of the differentiation between profane and sacred knowledge, modern scholars often refer to profane knowledge as sociocultural knowledge, also variously referred to as knowledge by acquaintance, commonsense knowledge, or everyday knowledge. This type of knowledge is often tied to a specific social group’s unexamined cultural beliefs, rituals, habitus, and ideological purposes which can be learned out of the classroom and in everyday life (Young, 2008; Rata, 2012; Winch, 2017).

In contrast to sociocultural knowledge which is socially constructed, disciplinary (sacred) knowledge is considered as the knowledge which has independently objective epistemic properties and which is professional, academic, rational, and powerful (Young, 2008; Rata, 2012; Beck, 2013; Vernon, 2020). Disciplinary knowledge has been created all along through human history and has been publicly scrutinized and contested over time and space so that it has its independent legitimacy and explanatory power of generalization and universalization (Rata, 2012; McPhail and Lourie, 2017). This type of knowledge is made up of abstract epistemic concepts which are immaterial and objective, with complex structures, and which are difficult to learn so they need to be taught in the classroom (Rata and Tamati, 2021). When abstract disciplinary knowledge enters the modern school disciplines and becomes contained in a school curriculum, it should be designed and altered to be concrete, visible, and available for teaching in the classroom (Rata and Tamati, 2021). This is also the aim of the CDC Model, that is, to design the abstract “conceptually integrated concepts” of the disciplinary knowledge into the concrete curriculum content for schooling (McPhail, 2020b, p. 394).

The mechanisms of incorporating disciplinary knowledge into the CDC Model are complex. To unpack the complexity and facilitate its application, the model creators dissected the model into four elements: curriculum proposition, content, competence, and evaluation (Rata, 2019, 2021b; McPhail, 2020a). The first element concerns how to design a curriculum proposition based on a given topic/theme, and how to extract key disciplinary concepts from that proposition for teaching. For example, when teaching the mathematical topic of “size,” an ECE teacher may create this curriculum proposition: “size refers to the length of something, which is often described by using two words: long and short.” From this proposition, the teacher may extract concepts in the discipline of mathematics such as “length,” “long,” and “short.”

The second element shows how to embed the extracted abstract disciplinary concepts into concrete curriculum content, such as stories, texts, and videos. For example, when teaching the concepts of “long” and “short,” a Mathematics teacher can draw a picture of two rulers or use two pencils with different lengths for students to compare. In this way, the teacher helps to materialize the abstract and invisible meaning of “long” and “short” into the concrete length of visible things. The materiality of immaterial disciplinary concepts often follows two criteria (Rata, 2021b). The first criterion is the *from-abstract-to-concrete* approach, referring to the sequencing of disciplinary concepts prior to the selection of curriculum content. Once the disciplinary concepts are selected, they are then sequenced per the second criterion – by following the *from-lower-to-higher-ordered-knowledge* approach (Rata, 2016). For example, an ECE teacher often teaches students lower-ordered concrete concepts such as “cat,” “dog,” and “sheep” before the higher-ordered abstract concept of “animal.” Also, students are often taught “letter” before “word” in language acquisition and “line segment” before “geometric figure” in mathematics.

The third element demonstrates how to teach the disciplinary concepts and content through pedagogic activities that help students develop the two resulting curriculum competencies: judgment and practical competence. Judgment competence denotes the ability to understand and explain the meaning of a disciplinary concept (knowledge-what) and to use it to explain why something is the case (knowledge-why). Practical competence refers to being able to put the acquired concept into practice (knowledge-how-to) (Rata, 2021b). *Knowing-what* and *knowing-why* lead to identifying *knowing-how-to*. In turn, *knowledge-how-to* is an illustration of *knowledge-what* and *knowledge-why* in practice (Rata, 2021b). For example, an ECE teacher often undertakes hands-on training to develop the numeracy of students by assisting them in understanding numbers (knowledge-what), differentiating a specific number from the others (knowledge-why), and using numbers to count things (knowledge-how-to).

The fourth element offers some evaluative methods to test whether students demonstrate the curriculum competencies after learning the disciplinary concepts and content. These methods include memorization (recall the meaning of a concept, knowing-what), explanation (using a concept to explain why something is the case, knowing-why), and application (putting a concept into practice, knowing-how-to). For instance, a language teacher asks students to recall the meaning of a word (memorization), to pronounce the word (application), and to tell the word from similar ones (explanation).

The conceptual structure of a discipline that is made up of disciplinary concepts provides the coherence device of the four elements of the CDC model (Muller, 2009). That is also the reason why disciplinary concepts permeate the CDC Model and link the four elements: curriculum proposition (from which a teacher extracts disciplinary concepts; the abstract and invisible meaning of curriculum knowledge), curriculum content (which materializes the disciplinary concepts; the concrete and visible content of curriculum knowledge), curriculum competencies (which demonstrate understanding and mastery of disciplinary concepts; what can be done with curriculum knowledge), and curriculum evaluation (which examines whether the disciplinary concepts were acquired, the outcomes of acquiring disciplinary concepts) (McPhail, 2020a). This also explains why this curriculum design method is named the Curriculum Design Coherence Model and why the CDC Model enables the promotion of disciplinary-knowledge-enriched teaching and learning.

## Research Design

This section introduces the research design and implementation. We first introduce how the CDC Model was introduced to a Chinese kindergarten, the research participatory kindergarten, to guide its kindergarten-based curriculum planning and teaching at the beginning of 2021. The kindergarten is a role model at the municipal level and ranks among the top five kindergartens in that city. Since the outset of 2021, the top five kindergartens in the city started to design kindergarten-based curricula that integrate the city’s cultural heritage and local educational resources. The curriculum practices of the five kindergartens are part of the local government’s educational agenda to develop the localized ECE curricula with municipal characteristics and to popularize the curricula throughout the city in the third decade of the 21st century.

The research participatory school is one of the five kindergartens which adopted the CDC Model in planning and developing its kindergarten-based curricula. The kindergarten has a total of 26 staff, including 21 academic staff. The 21 academic staff are teachers from the five ECE areas of health, language, society, science, and art. As prescribed in the national education policy, the five areas are compulsory in China’s ECE settings (MoE, 2012). Following the national policy, the kindergarten offers seven courses that embed the five areas (see Table 1). We provide detailed information about the 21 teachers as they played the main role in attending the training on the CDC Model, in developing the kindergarten-based curricula under the guidance of the CDC Model, and in using the CDC Model in their teaching practices. Therefore, they are also the main research participants of the study.

**TABLE 1:** The participants.

ECE area prescribed in the national policy	Courses offered by the kindergarten	Number of teachers	Average years of work experience	Academic degree
Health	Physical Education and Health	4	3.5	All bachelors
Language	Chinese and English	5	4	All bachelors
Society	Story	3	2	All bachelors
Science	Mathematics	5	4	4 Bachelors and 1 master
Art	Music and Fine Art	4	3	All bachelors

In 2021, the kindergarten conducted a total of six sessions of teacher training on the CDC Model, one session every 2 months. The duration of each session varied from 2 to 3 h. Of the six sessions, the first two focused on introducing the CDC Model and exemplifying its applications with specific curriculum design cases. The last four sessions were around the application of the CDC Model. The 21 teachers were invited to share their curriculum design cases which demonstrated the use of the CDC Model, followed by discussions of any comments, suggestions, and questions raised in the process.

Immediately following each training session, the kindergarten organized the 21 teachers to put the CDC Model into the kindergarten-developed curricula practices. Upon our empirical investigation, we found that the teachers had accomplished four tasks with the CDC Model as a guide. First, they determined the disciplinary concepts for the curriculum of each course contained in the 3-year kindergarten program, sequenced them following the from-concrete-to-specific order and from-lower-to-higher-ordered-knowledge approach, and allocated them into the specific courses used in the six respective semesters of the three academic years. Second, they configured specific curriculum content for the sequenced disciplinary concepts. Third, they formulated new standards of curriculum evaluation for teachers and students. Finally, they organized a teaching competition around the kindergarten-developed curricula, exploring effective ways to put the curricula into practice.

The study was conducted after the six training sessions and the teachers’ completion of the foregoing four curriculum-related tasks. As mentioned above, the 21 teachers all participated in planning the kindergarten-based curricula as major designers, developers, and implementors. To have them all involved in the research, upon having received the research ethics approval, and with their consent, we conducted a semi-structured focus group discussion with them to investigate the impact of the CDC Model on their curriculum designing and the following teaching practices. This method proved to be effective in researching the impact of the CDC Model on the 21 teachers’ teaching practices. In particular, this method helps us to observe the participants talking about the same topic from multiple perspectives and to collect data about their interactive dis/agreements or corrections that cannot be obtained from individual interviews (Hennink, 2014).

In this study, the focus group discussion started with a structured session for the review of the CDC Model. Then the opportunity was passed to the floor for the teachers to conduct a collective face-to-face discussion about the impact of the CDC Model on their teaching practices, lasting approximately 2 h. Given that running a focus group with 21 participants was very difficult to do, the participants were first divided into seven small groups according to the courses they were teaching at the moment, with some semi-structured questions (such as “What difficulties have you encountered in applying the CDC Model?,” “How do you overcome the difficulties?,” “In what ways have the CDC Model contributed to or hindered your teaching practices?”) provided for them to stimulate their discussion. After that, one teacher from each of the seven groups was invited to share the opinions on behalf of the group members. When all the viewpoints were collected from the seven groups, the 21 teachers were organized to conduct a free discussion on some of the opinions such as “What should be the starting point of the kindergarten-developed curricula: students’ experiences and life-worlds or teachers’ professional knowledge and understanding or others?,” “What is the most creative part in the use of the CDC Model?”

All the conversations were in Chinese to avoid any possible ambiguities or misunderstandings and at the same time to enable the participants to share more in-depth views by using their mother tongue. Furthermore, conducting the discussion in Chinese allowed us, as Chinese researchers, to “take advantage of [our] linguistic ability and cultural awareness” in the investigation (Sit, 2012, p. 32). We made digital audio recordings of the discussion and then transcribed them verbatim for substantial analysis (Nowell et al., 2017). The empirical study aims to answer the research question: To what extent and in what ways has the CDC Model exerted an influence on the ECE teachers’ teaching practices? The next section reports on the research findings, with data collected from the 21 teachers merged to protect their identity.

## Research Findings

The research finds the CDC Model has influenced the teaching practices of the 21 teachers mainly through (i) promoting their initiative for curriculum designing, (ii) forging the curriculum knowledge orientation, (iii) strengthening the conceptual structure of the kindergarten-developed curricula, and (iv) enhancing the coordination among the curricula of the seven courses provided by the kindergarten.

### Promotion of Initiative for Curriculum Designing and Teaching

Professional teaching and learning require teachers to take the initiative in designing and teaching curricula (Gao, 1998). However, the focus group discussion reveals that the teachers’ curriculum designing and teaching initiative was suppressed for a long time. Prior to the kindergarten-developed curricula, the teachers were asked to use the nationally prescribed curricula and teaching materials. Curriculum development was regarded as a highly specialized task of the experts because its creation, implementation, and evaluation were monopolized by them to a great extent. As practitioners, the 21 teachers merely played a supporting role in the implementation of the prescribed curricula. Under such conditions, resistance or even hostility toward mandated education may be generated from the teachers because the “true teacher voice” was repressed (Kragler et al., 2008, p. 547).

The long-term passive curriculum implementation also to some extent led to the loss of the teachers’ curriculum initiative. The group discussion shows that the majority of them abandoned the unique and innovative ideas for curriculum design and teaching that they had had at the outset of their careers as ECE teachers, and went back to the official curricula because they were judged as having deviated from the prescribed curriculum content and evaluation methods. In addition, the official curricula provided them with ready-made materials such as pedagogic plans, goals, preparations, procedures, processes, tips, and textbooks. In the discussion, many of them expressed that they had become substantively dependent on the national curricula and the easy-to-get supporting curriculum resources, losing their initiative in designing curriculum.

Being deprived of the curriculum initiative for a long time, the teachers felt like *fish out of water* at the start when they were assigned the task to develop the kindergarten-based curricula. This task stagnated at the outset because the teachers did not have a solid knowledge foundation in curriculum design (most of them had only a bachelor’s degree) and thus lacked professional knowledge of curriculum development. In addition, they obtained little professional training on curriculum planning. As a result, the task returned the right of curriculum development to the teachers, but it brought them huge psychological and professional challenges and pressures.

Fortunately, the training on the CDC Model reduced this pressure and promoted their initiative in curriculum development. They expressed in the group discussion that the CDC Model had guided them in designing the kindergarten-based curricula, which changed their role from mere curriculum users to curriculum designers and executors. This increase in curriculum initiative further stimulated their pedagogical agency to make critical reflections on the curricula used for the seven courses offered by the kindergarten. For example, some music teachers in the discussion mentioned that they had incorporated the disciplinary concept of “noise” into the Music Curriculum. For them, the distinction between music and noise should have been the very first lesson contained in the course. This also demonstrates the growth of their professionalism because one indispensable characteristic of teachers’ professional development lies in their ability to make critical reflections (Lehrer, 2013).

The CDC Model also contributed to the improvement of the teachers’ pedagogy by enhancing the professional cooperation among them, which further promoted their initiative in curriculum implementation. According to the teachers, the kindergarten organized a pedagogic competition called *One Lesson, Three Classes* for teaching the kindergarten-developed curricula. In the competition, every three teachers were invited to design and teach the same lesson contained in the curricula they had jointly designed and the other teachers were asked to join the lesson as commentators. After each lesson, the teacher commentators would evaluate the lesson, the pedagogic strategies, activities, and design. The lesson lecturer was asked to do a self-reflection on the lesson. The CDC Model was adapted to offer the criteria for the evaluation and self-reflection.

According to the teachers, the competition improved their teaching initiative. For now, when designing a lesson, the majority of them have become used to asking themselves a series of independent and critical questions, such as, what disciplinary concept does the lesson address? What content is used to teach the concept? Is the content familiar to students? Can the content help students understand the concept and develop corresponding curriculum competencies? The growing responsibility for curriculum implementation and their desire to provide quality lessons to students indicated the growth of the teachers’ pedagogical leadership and initiative in curriculum designing and teaching (Heikka et al., 2016; Ukkonen-Mikkola and Fonsén, 2018; Fonsén and Ukkonen-Mikkola, 2019). Moreover, the CDC Model enhanced the professional cooperation and communication between the teachers, and these collaborative works are supportive of teachers’ professional development (Cherrington and Thornton, 2013; Cotton, 2013).

### Orientation Toward Curriculum Knowledge

The CDC Model helps to eliminate the differences between the teachers and promotes an orientation toward disciplinary knowledge in their teaching practices. This is the case because the definition of curriculum teaching is often vague in the ECE context (Kangas et al., 2021). The group discussion showed that disputes had existed between the teachers over planning the content and pedagogy of the kindergarten-based curricula before the training on the CDC Model.

Some teachers believed that the kindergarten-developed curricula should proceed from students’ experiences and life-worlds. They argued that teaching should be student-centered. For them, the ECE classrooms in the past were too much around the official curricula and the pursuit of the prescribed skills, neglecting students’ learning interests. Therefore, they argued that the kindergarten-developed curricula should not address substantive literate or numeracy skills, but enable students to have a basic understanding of symbols, to attract their learning interest, and to allow them to study in a more relaxed environment – that is enough.

However, other teachers thought that the kindergarten-developed curricula should be established on the teachers’ professional knowledge, rather than the interests of students. For them, only when the teachers design and implement the curricula with their professional understanding and experience of the students can they truly guide the students to learn the curricula. As for pedagogy, they held no brief for a student-led teaching approach which, according to them, often overestimates students’ cognitive ability, life experience, and social skills. A lesson called *The Little Sheep is Angry* for year-two students was mentioned as an example by a teacher in the discussion to demonstrate the disadvantages of a student-led pedagogy. The teacher used a student-led pedagogy by inviting the students to listen to the story, analyzing the causes and consequences of the little sheep’s anger, and then answering the question: “How can the sheep relieve its anger?” The teacher constantly guided the students to answer the question without bringing up any of his ideas. In the discussion, the other teachers who audited the class rated it to be not successful because year-two students were too young to express their limited views in complete and coherent words and thus were not suited for a student-centered pedagogy.

The CDC Model settled the teachers’ differences in curriculum design and pedagogy. According to the discussion, they now spontaneously agree that neither students’ life-worlds nor teachers’ professional understanding of students should be the curriculum basis, but it should be disciplinary knowledge. In addition, professional teaching should center on neither teachers nor students, but on the competencies developed based on the acquisition of curriculum knowledge. For example, following the CDC Model, the aforementioned teacher improved his lesson design. The teacher still adopted a student-led pedagogy but he brought three pictures of a sheep listening to music, a sheep doing housework, and a sheep running in the park, into the lesson as hints to guide the students to come up with their solutions to anger. The teacher became much clearer that the lesson aimed to teach the students the disciplinary concept of “anger” and to develop their competencies to overcome being angry. This increase in the teachers’ pedagogical competence suggested how the CDC Model had empowered them as professional designers and agents of both curriculum and pedagogy (Fonsén and Ukkonen-Mikkola, 2019).

### Strengthening the Conceptual Structure of the Curricula

The application of the CDC Model attributes to strengthening the conceptual structure of the kindergarten-based curricula used for the seven courses in the kindergarten. That is, the CDC Model has helped the teachers to establish the kindergarten-developed curricula by following the *from-the-concrete-to-abstract* order and the *from-lower-to-higher-ordered-knowledge* structure mentioned above. For example, the mathematics teachers interviewed mentioned in the group discussion that, when making the Mathematics Curriculum for teaching the concept of “geometric figure,” the curriculum focused on teaching the figures of circles, squares, and triangles to ECE year-one students by aligning these figures with real objects such as car tires, floor tiles, and kites. The curriculum for ECE year-two students was about knowing the more complex figures of rectangles, ellipses, trapezoids, and rhombus by mastering their basic and unique characteristics such as corners and sides. The curriculum for ECE year-three students highlighted cultivating students’ competence to draw the foregoing figures by the descriptions of their characteristics.

The teachers believed the disciplinary concept of “geometric figures” helped to achieve the conceptual structure of the Mathematics Curriculum used in the three academic years. The CDC Model takes the *from-the-concrete-to-abstract* order and *from-lower-to-higher-ordered-knowledge* approach in curriculum concept sequence and progression. Therefore, the teachers had considered and designed the multimodalities of the disciplinary concept of “geometric figure” by linking it to students’ cognitive characteristics of processing mathematical concepts. To be specific, and according to the discussion, 3- or 4-year-old students (ECE Year One) have a weaker perception of geometric figures, and their eyes can only focus on the interior rather than the outline of a figure, therefore they cannot accurately identify the figure. Because of this, the curriculum was determined to align simple figures with real objects to establish students’ geometric awareness. However, students around the age of five (ECE Year Two) have better perception ability and begin to notice the typical parts of a figure. Therefore, the curriculum for year-two students focused on recognizing more complex figures *via* their unique characteristics. For year-three students (around the age of six), their perception ability is further developed, which allows them to form a cognitive pattern that can control the direction and range of their eye movements along the outline of a figure as if making a typical model of the figure in their mind for recognition of it. Therefore, the curriculum was designed for students to draw a figure by the descriptions of its characteristics. The discussion indicated that it was the CDC Model that had guided the teachers to strengthen the conceptual structure of the kindergarten-developed curricula.

### Enhancement of the Coordination Between Different Curricula

The group discussion also indicated that the CDC Model facilitated and enhanced the coordination among the kindergarten-developed curricula. We use the term “coordination” to refer to the linkage of the same disciplinary concept contained in the curricula of the seven different courses. According to the discussion, curriculum coordination was a collective intellectual invention that came from the teachers’ discussions and reflections.

In the group discussion, the teachers mentioned the teaching of the concept “cat,” which may be a good example to explain what is curriculum coordination as theorized by us. While developing the kindergarten-based curricula, they had arranged the teaching sequence and assigned different focuses in each of the curricula of the seven different courses offered by the kindergarten to teach the concept. To be specific, to teach the concept of “cat,” the Fine Art Curriculum focuses on using pictures to guide students in understanding the meaning of “cat” first. Then, the Chinese and English Curricula focus on reading and writing the word “cat.” After that, the Mathematics Curriculum develops students’ numeracy ability by using questions like “How many cats are there in the picture?” and cultivates students’ geometric ability *via* guiding students to draw a cat using shapes like circles, triangles, and rectangles. The Story Curriculum highlights the characteristics and personality of cats by using fables. The Music Curriculum embeds the “miaow” sound of the cat into lyrics for students to sing. The Physical Education and Health Curriculum incorporates the learning of the cat into games called “cat chases mouse.”

According to the teachers, the coordination between the curricula used for the parallel courses offered by the kindergarten not only provided them with the means to touch the five academic areas of health, language, society, science, and art prescribed in the national ECE policy (MoE, 2012) but also strengthened their sensitivity to curriculum resources available around them. This also witnessed the value of quality teacher education in enhancing the professional competencies of teachers (Ukkonen-Mikkola and Fonsén, 2018; Fonsén and Ukkonen-Mikkola, 2019).

## Discussion and Conclusion

In this section, we conclude the article by discussing the inherent relationship between the CDC Model, its implementation, and the outcomes as observed in the empirical study. We find that the relationship is embedded in how the CDC Model has contributed to the three independent traits of teachers’ professionalism in curriculum designing and teaching as theorized by Gao (1998). Therefore, we forged our discussion around the three traits.

The first trait addresses the professionalism that is built upon professional knowledge and theories. The CDC Model was created based on curriculum theories (Rata, 2019, 2021b; McPhail, 2020a). The training on the CDC Model, its four elements, and the linkage between them enriched the teachers’ knowledge bank on curriculum design which in turn increased their professionalism in designing the kindergarten-based curricula. As a consequence, the task to develop the curricula offered a solid epistemological basis for them to practice their knowledge and skills in curriculum design, which further fostered their professionalism. As noted by Fonsén and Ukkonen-Mikkola (2019), “The benefits of continuous professional learning enhance reliance and support the motivation to gain further professional knowledge” (p. 185). The opportunities which allow the teachers to link their professional knowledge to their teaching practices also constitute one “core [feature] that [characterizes] effective professional development” of ECE teachers (Zaslow et al., 2010, p. ix).

This also reflects why an increasing number of studies call for greater attention to be paid to ECE teachers’ professional development by addressing the important role of teacher knowledge and content-rich pedagogy (e.g., Cervetti et al., 2012; Neuman and Kaefer, 2018; Neuman and Danielson, 2020). For example, Breffni (2011) suggests that professional curriculum training has proven to be effective in facilitating and improving prekindergarten teachers’ knowledge and practices in the United States. McLachlan et al. (2017) find that by engaging with professional development programs that address teacher knowledge and skills, teachers’ curriculum practices can be improved effectively in New Zealand.

The second trait suggests that professionalism often has a unique function to perform. The CDC Model performed its unique function of assisting teachers in understanding the importance of disciplinary concepts and forging their teaching practices toward curriculum knowledge. The discussion revealed that the teachers agreed that there should be a disciplinary concept behind their teaching practices. As they exemplified in the discussion, teaching students how to perform on a balance beam can help them understand the concept of “balance.” This also resonates with Chan et al.’s (2009) research where the teachers used waste as materials and dolphins as symbols to teach children the disciplinary concepts of “recycling” and “harmony.”

The teachers’ re-identification and recognition of the central role of disciplinary knowledge in their teaching practices *via* the training and utilization of the CDC Model may also help them teach disciplinary knowledge, which Young (2008) calls *powerful knowledge*. This is because teachers’ interest and motivation in adjusting their teaching practices were stimulated and increased once they presented high fidelity to the curriculum and its underpinning philosophy (Lieber et al., 2009). In addition, a clear understanding of the educational core and decision-making process in the teaching practices is also essential for teachers to serve children and their families, especially in the contemporary period (Gordon et al., 2016).

The third trait denotes that professionalism is not subject to the control of external forces and is reliable for teachers to make autonomous professional judgments. The CDC Model helped the teachers make independent decisions in their teaching practices. The foregoing coordination between the kindergarten-developed curricula of the seven courses, as the collaborative creation of the teachers, is a good example to illustrate this. The teachers also expressed their growth in making professional judgments by overcoming independently the two major difficulties that they encountered in the application of the CDC Model.

The first difficulty was how to design a good curriculum proposition. According to the CDC Model, the design departure is a given topic; a curriculum proposition is designed based on the given topic; disciplinary concepts, usually more than one, are extracted from the curriculum proposition; curriculum content is designed to teach the concepts (McPhail, 2020a). To this end, the purpose of designing a curriculum proposition is to teach multiple disciplinary concepts contained in one lesson. However, the lessons contained in the kindergarten-based curricula were not that complex and each lesson usually touched on only one disciplinary concept. To this end, there is no need to design a proposition and then extract only one knowledge concept from it for teaching. In their practices, the teachers changed the curriculum design departure from topics to disciplinary concepts. They first examined the history, major debates, and *status quo* of a discipline to sort out the concepts that students need to know about the discipline, and then materialized the concepts into appropriate and specific content and topics. This avoids the procedure of making curriculum propositions, thus overcoming the first difficulty. Their practices proved this solution was useful.

According to the teachers, determining the concepts first before the content also offered them ways to overcome the second difficulty in using the CDC Model: the curriculum concept and content were sophisticatedly intertwined, sometimes making it difficult to distinguish one from the other. By selecting concepts and sequencing them before preparing the content, the teachers can ascertain that the curriculum concepts follow a *from-lower-to-higher-ordered-knowledge* sequence that promotes students’ conceptual progression (Rata, 2016). In this way, even if sometimes the content and concepts were intertwined, the internal disciplinary structure of the curriculum knowledge remains unchanged.

To conclude, the CDC Model enables the teachers to become the curriculum designers, in Moss’s (2008) words, “democratic and reflective professionals” in curriculum development (p. 125) by promoting their curriculum initiative, forging curriculum knowledge orientation, strengthening the conceptual structure of the kindergarten-developed curricula, and enhancing the coordination between parallel curricula. This positive influence also helped them bridge the disagreement on curriculum content and pedagogy and overcome some difficulties in using the CDC Model independently.

It is important to treat curriculum design and implementation as educational practices to do *with* ECE teachers, rather than *to* them, and to make them the active agents in curriculum-based endeavors (Cherrington and Thornton, 2013; Baker, 2017). The study shows the value of the CDC Model and the model-based curriculum practices in helping teachers overcome the traditional trainer-directed teacher training with limited follow-up on learning practices that thus reduces the training effectiveness (Karagiorgi et al., 2008). The study also has implications for conducting continuous and regular further professional training for ECE teachers because they “deserve the highest quality professional learning to support the implementation of new instructional materials and curriculum” (Short and Hirsh, 2020, p. 2). Most importantly, the study has implications for revitalizing the value of disciplinary knowledge in teaching and learning that has relevance to a broad international audience of ECE agents, including policymakers, teachers, stakeholders, practitioners, researchers, and parents (Rata, 2021a).

## Data Availability Statement

The original contributions presented in this study are included in the article/supplementary material, further inquiries can be directed to the corresponding author.

## Ethics Statement

The studies involving human participants were reviewed and approved by the Ethics Committee of School of Humanities and Foreign Languages, Xi’an University of Posts and Telecommunications. Written informed consent to participate in this study was provided by the participants.

## Author Contributions

XT, LB, and TL contributed to the conception and design of the study, conducted the data analysis, and wrote the first draft of the manuscript. YG organized the database and validated the data analysis. All authors contributed the to manuscript revision, read, and approved the submitted version.

## Conflict of Interest

The authors declare that the research was conducted in the absence of any commercial or financial relationships that could be construed as a potential conflict of interest.

## Publisher’s Note

All claims expressed in this article are solely those of the authors and do not necessarily represent those of their affiliated organizations, or those of the publisher, the editors and the reviewers. Any product that may be evaluated in this article, or claim that may be made by its manufacturer, is not guaranteed or endorsed by the publisher.
